# Orexin deficiency and cardiometabolic risk in narcolepsy: mechanistic pathways and therapeutic implications

**DOI:** 10.1152/function.90001.2026

**Published:** 2026-12-01

**Authors:** Anant Shukla, Jacqueline H. Geer, Brandon Nokes, Brian S. Wojeck

**Affiliations:** ^1^Section of Pulmonary, Critical Care and Sleep Medicine, Yale School of Medicine, New Haven, Connecticut, United States; ^2^Yale Center of Sleep Medicine, Yale New Haven Hospital, New Haven, Connecticut, United States; ^3^Sleep Medicine Section, VA San Diego Healthcare System, San Diego, California, United States; ^4^Division of Pulmonary, Critical Care, Sleep Medicine and Physiology, University of California San Diego, La Jolla, California, United States

**Keywords:** cardiometabolic risk, insulin resistance, narcolepsy type 1, orexin, orexin receptor agonist

## Abstract

Narcolepsy type 1 (NT1) results from autoimmune destruction of orexin-producing neurons in the lateral hypothalamus. While traditionally viewed as a sleep disorder, accumulating evidence indicates that orexin deficiency produces widespread cardiometabolic consequences—including obesity, insulin resistance, dyslipidemia, metabolic-associated steatotic liver disease, and major adverse cardiovascular events—that cannot be fully explained by weight gain or medication effects alone. This review examines four overlapping mechanisms through which orexin deficiency drives cardiometabolic risk. First, loss of orexin disrupts the coupling of hunger drive with arousal and energy expenditure. Orexin neurons interact with neuropeptide Y (NPY)/agouti-related peptide (AgRP) and pro-opiomelanocortin (POMC) circuits and project to reward and cognitive control pathways; their absence impairs satiety signaling, blunts hedonic reward processing, and uncouples food intake from physical activity. Second, orexin has direct peripheral metabolic effects—enhancing beta-cell function, promoting adipocyte insulin sensitivity, and suppressing hepatic gluconeogenesis. Loss of these actions promotes insulin resistance, atherogenic dyslipidemia, and hepatic steatosis. Third, disrupted sleep architecture and autonomic dysfunction compound metabolic risk. Orexin loss produces state-dependent dysautonomia—reduced resting sympathetic drive with inadequate parasympathetic compensation during sleep—contributing to nondipping blood pressure and elevated cardiovascular risk. Disease-specific barriers, including cataplexy avoidance, further limit physical activity. Fourth, the autoimmune inflammatory process may contribute to systemic metabolic inflammation, though the extent of ongoing inflammation after neuronal loss remains uncertain. These mechanisms are interdependent and mutually reinforcing. Selective orexin receptor 2 agonists such as oveporexton offer the first opportunity to restore lost orexin signaling. Whether this translates into cardiometabolic improvements warrants systematic evaluation in future trials.

## Graphical abstract

**Figure:**
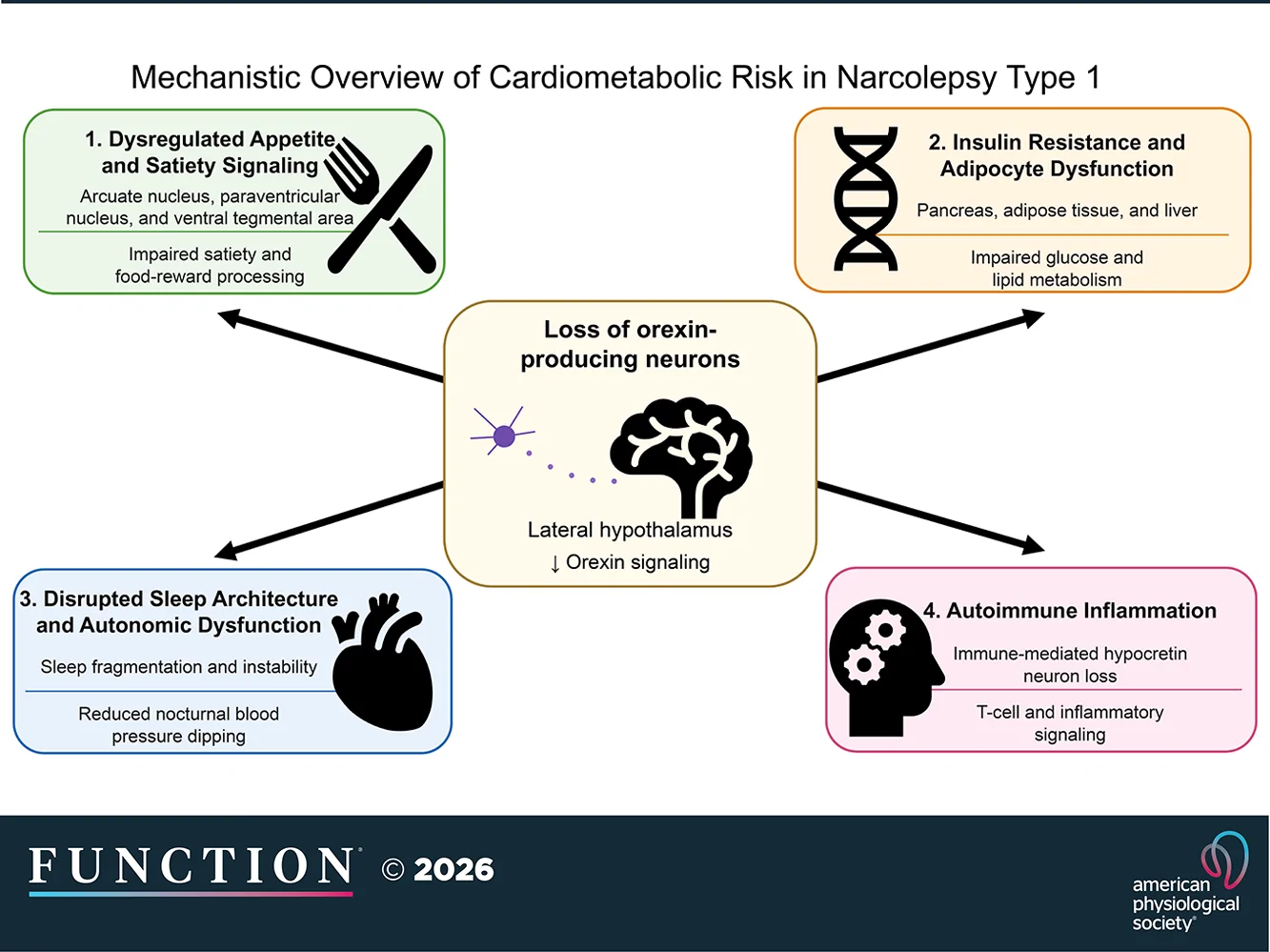

## INTRODUCTION

Orexin deficiency in narcolepsy type 1 contributes to cardiometabolic risk through multiple interacting pathways—including dysregulated satiety signaling, insulin resistance, sleep disruption, and autoimmune inflammation. These mechanisms overlap and reinforce each other over time rather than acting independently. This review examines the physiological basis for this increased risk and considers the therapeutic implications of emerging orexin-based therapies.

Orexin-A and orexin-B (also referred to as hypocretin-1 and -2, respectively) are hypothalamic neuropeptides originally identified in the late 1990s in the context of feeding behavior and arousal (1, 2). Their discovery, by two independent groups approaching the problem from different directions, ended up reshaping how sleep regulation was understood (1, 2).

These peptides are produced by a small group of neurons in the lateral hypothalamus, estimated at roughly 70,000 cells. Despite that, their projections extend widely across the central nervous system. Through these connections, the orexin system influences sleep-wake regulation, energy balance, reward pathways, autonomic control, and neuroendocrine signaling (3). In practical terms, the orexin system behaves less like a discrete circuit and more like a coordinating network that links behavioral state with metabolic demand.

Orexins act via two G-protein-coupled receptors, orexin-1 receptor (OX1R) and orexin receptor 2 (OX2R), which have overlapping but not identical patterns of expression in both central and peripheral tissues (4). OX1R shows higher affinity for orexin-A, whereas OX2R binds both peptides with similar affinity (4). Functionally, OX2R appears to play the dominant role in maintaining wakefulness, whereas OX1R is more closely tied to reward and feeding-related behaviors (5).

Importantly, orexin receptors are present in peripheral tissues such as pancreatic islets, adipose tissue, adrenal glands, and parts of the gastrointestinal tract (4). Orexin signaling is thus not confined to the brain and contributes to systemic metabolic regulation.

The orexin system has a context-dependent role in glucose regulation. During hypoglycemia, it helps maintain euglycemia by promoting glucose mobilization, epinephrine release, and feeding behavior. Under hyperglycemic conditions, it enhances insulin sensitivity via increased GLUT 4 expression (6). This kind of bidirectional behavior suggests that orexin helps maintain metabolic balance rather than driving metabolism in one direction. Thus, when orexin is deficient, what is disrupted may be state-dependent metabolic coordination rather than just sleep itself.

The clinical data broadly support this idea. Meta-analyses show increased prevalence of obesity, diabetes, hypertension, and dyslipidemia in patients with narcolepsy compared with matched controls (7). Metabolic syndrome has been reported in ∼30% of narcolepsy patients overall, and up to 59% of those with NT1. Prediabetes and nondipping blood pressure patterns, which are both associated with adverse cardiovascular outcomes, frequently cooccur (8).

Large retrospective cohort studies reinforce this pattern. In one study including over 22,000 patients with narcolepsy, higher rates of hypertension, hyperlipidemia, diabetes, metabolic-associated steatotic liver disease (MASLD), and major adverse cardiovascular events were observed compared with matched controls (9). These associations held even after adjusting for treatment effects and were present in younger patients, suggesting that cardiometabolic dysfunction starts relatively early in the disease course (9).

**Figure 1.**
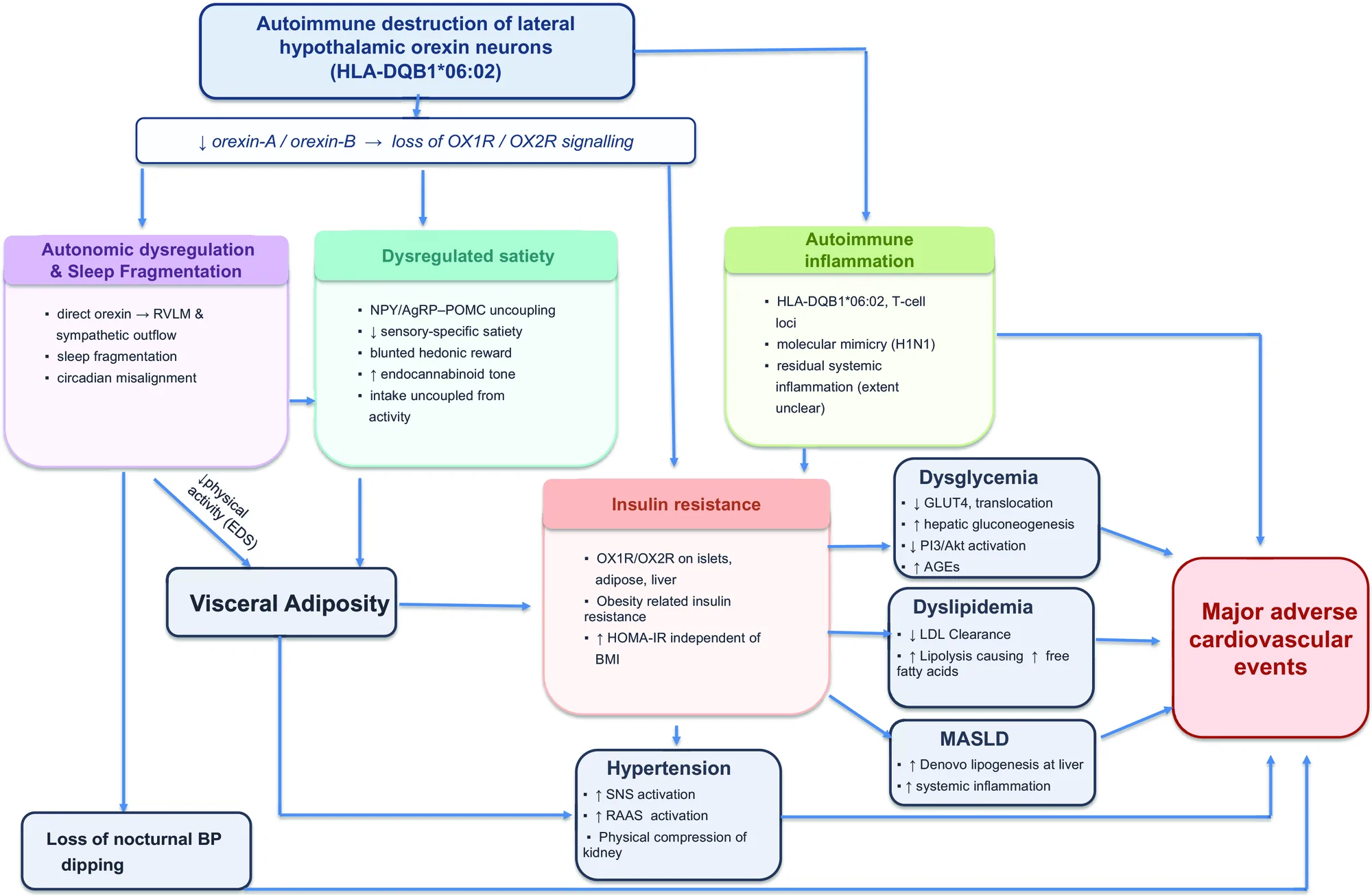
Mechanistic pathways linking orexin deficiency to cardiometabolic risk in narcolepsy type 1. Autoimmune destruction of lateral hypothalamic orexin neurons (associated with HLA-DQB1*06:02*) eliminates orexin-A/B signaling through OX1R and OX2R, triggering four interconnected upstream pathways: *1*) autonomic dysregulation and sleep fragmentation, *2*) dysregulated satiety, *3*) autoimmune inflammation, and *4*) insulin resistance. Dysregulated satiety and autonomic dysregulation (via reduced physical activity from excessive daytime sleepiness) promote increased visceral adiposity, which in turn exacerbates insulin resistance in a feed-forward cycle. Autonomic dysregulation also drives loss of nocturnal blood pressure dipping. Insulin resistance feeds directly into downstream cardiometabolic consequences—dysglycemia, dyslipidemia, MASLD, and hypertension—while autoimmune inflammation contributes both to insulin resistance and directly to major adverse cardiovascular events. Loss of nocturnal BP dipping contributes to both hypertension and cardiovascular risk independently. All four downstream consequences converge on elevated risk of major adverse cardiovascular events, which are present even in younger patients. AGEs, advanced glycation end-products; AgRP, agouti-related peptide; BMI, body mass index; BP, blood pressure; EDS, excessive daytime sleepiness; GLUT4, glucose transporter type 4; H1N1, influenza A virus subtype; HLA, human leukocyte antigen; HOMA-IR, homeostatic model assessment of insulin resistance; LDL, low-density lipoprotein; MACE, major adverse cardiovascular events; MASLD, metabolic dysfunction–associated steatotic liver disease; NPY, neuropeptide Y; OX1R, orexin receptor 1; OX2R, orexin receptor 2; PI3K/Akt, phosphoinositide 3-kinase/protein kinase B; POMC, pro-opiomelanocortin; RAAS, renin-angiotensin*-*aldosterone system; RVLM, rostral ventrolateral medulla; SNS, sympathetic nervous system.

Taken together, narcolepsy can be viewed as a multisystem disorder rather than a purely sleep-related condition. The following sections look at four overlapping mechanisms—dysregulated hunger and satiety signaling, insulin resistance, sleep disruption, and autoimmune inflammation—that may help explain this increased cardiometabolic risk. Figure 1 provides a schematic overview of these interconnected mechanisms, and Table 1 summarizes the supporting evidence for each.

**Table 1.** Summary of key evidence supporting the four mechanistic pathways linking orexin deficiency to cardiometabolic risk in NT1

Mechanism	Key Finding	Population/Model	References
Dysregulated appetite and satiety			
Impaired sensory-specific satiety	NT1 patients failed to shift food preference after satiation; consumed more calories ad libitum	Human (NT1 vs. controls)	van Holst 2016 (10)
Blunted hedonic reward signaling	VTA and nucleus accumbens responses blunted during reward tasks on fMRI	Human (unmedicated NT1)	Ponz 2010 (11)
Enhanced cognitive food-cue responses	Increased vmPFC activation to food cues predicted snack intake; not seen in idiopathic hypersomnia	Human (NT1 vs. IH)	van Holst 2018 (12)
Weight gain despite reduced intake	Orexin-KO mice gained weight with increased fat mass and hepatic steatosis despite eating less	Mouse (orexin-KO)	Devère 2025 (13)
Insulin resistance and adipocyte dysfunction			
Elevated HOMA-IR independent of BMI	Insulin resistance present early in pediatric narcolepsy, not explained by body weight	Human (pediatric NT1)	Zhang 2023 (14)
Orexin supports beta-cell function	Orexin-A enhances glucose-stimulated insulin secretion via PI3K/Akt/ERK and mTOR pathways	In vitro (INS-1E, rat insulinoma)	Skrzypski 2016 (15); Chang 2019 (16)
Orexin deficiency → MASH and HCC	Orexin-deficient mice on high-fat diet developed MASH with fibrosis; orexin supplementation attenuated changes	Mouse (orexin-deficient)	Tsuneki 2022 (17)
Impaired BAT thermogenesis	Orexin-null mice show lower energy expenditure despite similar caloric intake	Mouse (orexin-null)	Milbank 2019 (18); Sellayah 2011 (19)
Disrupted sleep and cardiovascular			
Sleep fragmentation	Reduced slow-wave sleep, more frequent awakenings, and sleep instability	Human (NT1)	Zhang 2021 (20); Maski 2022 (21)
No difference in energy expenditure	Calorimetry and doubly labeled water showed similar energy expenditure in NT1 vs. controls	Human (NT1 vs. controls)	Donjacour 2025 (22)
Loss of nocturnal BP dipping	Non-dipping blood pressure in a substantial proportion of NT1 patients	Human (NT1)	Zhang 2026 (8); Jennum 2021 (23)
Autonomic dysfunction	Increased sympathovagal imbalance during sleep; blunted cardiovascular reflexes	Human (NT1)	Grimaldi 2014 (24); Donadio 2014 (25); Aslan 2019 (26)
Autoimmune inflammation			
HLA-DQB1*06:02 association	Strong genetic association with NT1 across populations	Human (genetic studies)	Liblau 2024 (3); Luo 2018 (27)
Cross-reactive T cells	T cells recognizing both influenza antigens and hypocretin peptides identified	Human/in vitro	Luo 2018 (27)
Autoreactive T cells in blood and CSF	CD4^+^ and CD8^+^ T cells targeting hypocretin antigens detected in peripheral blood and CSF	Human (NT1)	Latorre 2018 (28)

References are given as first author, year, and reference-list number; additional supporting citations are provided in the corresponding sections of the text. BAT, brown adipose tissue; BMI, body mass index; BP, blood pressure; CSF, cerebrospinal fluid; fMRI, functional magnetic resonance imaging; HCC, hepatocellular carcinoma; HOMA-IR, homeostatic model assessment for insulin resistance; IH, idiopathic hypersomnia; MASH, metabolic associated steatohepatitis; NT1, narcolepsy type 1; vmPFC, ventromedial prefrontal cortex; VTA, ventral tegmental area.

### Mechanism 1: Dysregulated Appetite and Satiety Signaling

Orexin deficiency may also contribute to cardiometabolic risk by altering appetite, satiety, and food-related decision-making. This mechanism is distinct from insulin resistance, sleep disruption, and autoimmune inflammation because it focuses on how the brain regulates food intake itself, including hunger signaling, satiety perception, and reward-based eating.

### Hypothalamic Appetite Signaling

Orexin neurons reside in the lateral hypothalamus, anatomically distinct from the melanocortin system, which is organized around the arcuate nucleus and paraventricular nucleus of the hypothalamus. Nonetheless, orexin interacts directly with both components of this circuit. In the arcuate nucleus, orexin-A preferentially activates NPY/AgRP neurons through OX1R-coupled calcium signaling while suppressing POMC neuron activity through mechanisms involving both OX1R and OX2R, producing effects that are largely reciprocal to leptin (29–33). Synaptic connections between orexin neurons and NPY cells in the arcuate nucleus have been demonstrated in both rodent and primate hypothalamus, suggesting this interaction is conserved and functionally significant (34). Orexin-A signaling also reaches the paraventricular nucleus, where it promotes food intake through the agouti-related peptide (AgRP)/NPY pathway (35). Importantly, orexin is an orexigenic neuropeptide that normally couples hunger drive with arousal and energy expenditure, linking the motivation to eat with the sympathetic activation needed to seek food and burn energy. When orexin neurons are destroyed in NT1, hunger persists through intact orexigenic pathways (NPY/AgRP, ghrelin), but the corresponding drive toward physical activity, sympathetic tone, and thermogenesis is lost. This uncoupling of intake from expenditure likely contributes to the positive energy balance and weight gain observed in narcolepsy.

### Satiety, Reward Processing, and Food Choice

Human studies provide important evidence that orexin deficiency affects satiety and food-related behavior. van Holst et al. (10) showed that patients with NT1 have impaired sensory-specific satiety. After eating a specific food to fullness, healthy controls naturally shifted their preference toward different foods, whereas patients with NT1 continued selecting the same food and consumed substantially more calories during ad libitum snacking. This suggests that abnormal orexin signaling can disrupt the normal integration of satiety signals with reward-driven food choice.

Orexin neurons in the lateral hypothalamus also project directly to the ventral tegmental area (VTA), where they potentiate dopamine release in the nucleus accumbens through OX1R signaling, forming the hedonic reward circuit for food (36, 37). In NT1, this circuit appears functionally impaired. A functional magnetic resonance imaging (fMRI) study in patients with unmedicated NT1 found that VTA activity was not modulated during reward anticipation, and nucleus accumbens responses were blunted during reward delivery (11). Clinically, patients with NT1 show higher rates of binge eating and impulsivity, consistent with disrupted reward processing (38).

Separately, the cognitive control pathway also appears affected. Patients with NT1 show enhanced ventromedial prefrontal cortex (vmPFC) responses to food cues, with vmPFC activity predicting spontaneous snack intake—and this effect was not seen in patients with idiopathic hypersomnia, suggesting it is specific to orexin deficiency rather than sleepiness alone (12).

Together, these findings suggest that orexin loss disrupts both the reward valuation of food and the executive control over food-seeking behavior.

### Endocannabinoid-Mediated Satiety Suppression

Orexin may also influence appetite through endocannabinoid pathways. Morello et al. (31) described a mechanism in which orexin-A signaling in POMC neurons stimulates production of 2-arachidonoylglycerol, which then acts through cannabinoid-1 (CB1) receptors to suppress POMC transcription and reduce alpha-melanocyte-stimulating hormone (α-MSH) production. Since POMC and α-MSH are key satiety signals, this provides a plausible molecular pathway by which orexin signaling can blunt satiety. This pathway is important because it connects orexin biology directly to appetite regulation at the cellular level, rather than relying only on behavioral observations.

### Weight Gain Despite Reduced Intake in Orexin Deficiency

A key observation in orexin biology is that orexin-deficient animal models may eat less than controls but still gain weight. Devère et al. (13) showed that orexin-KO mice developed increased body weight, greater fat mass, impaired glucose tolerance, reduced insulin secretion, and hepatic steatosis despite reduced food intake in males. Similarly, González et al. (39) demonstrated that orexin neuron activity normally falls rapidly at the onset of eating and remains suppressed during consumption, suggesting that orexin neurons participate in the timing and control of feeding behavior. These findings imply that weight gain in orexin deficiency cannot be explained by caloric intake alone. Reduced energy expenditure, altered nutrient handling, impaired insulin secretion, and abnormal satiety signaling may all contribute.

More explicitly, daytime sleepiness lowers habitual activity in NT1, which promotes visceral adiposity and insulin resistance independent of caloric intake; because orexin normally couples arousal with locomotor drive and energy expenditure, its loss reduces the drive to move while orexigenic appetite signaling remains intact, widening the mismatch between energy intake and expenditure.

### Appetite Hormones: Mixed Human Data

Human studies of appetite hormones in narcolepsy have been inconsistent. Kok et al. (40) reported reduced plasma leptin levels and loss of normal circadian leptin rhythmicity in hypocretin-deficient patients. However, Donjacour et al. (41) found no significant difference in 24-h ghrelin or leptin levels when patients were carefully matched for body mass index (BMI), and a meta-analysis (7) did not show a consistent pooled leptin difference. Huda et al. (42) reported unchanged ghrelin but elevated obestatin, which correlated with autonomic dysfunction.

These mixed results regarding NT1 and its influence on the hormonal modulation of appetite are not surprising. Appetite hormones are influenced by body composition, autonomic tone, sleep timing, medications, and disease duration. Therefore, appetite dysregulation in NT1 is probably not explained by a single hormone abnormality.

Taken together, the evidence from impaired sensory-specific satiety, altered hormonal profiles, and disrupted orexin-mediated reward signaling suggests that orexin deficiency may alter not only metabolism and sleep but also how the brain registers satiety and responds to food cues. Appetite dysregulation therefore represents one pathway linking narcolepsy to obesity and cardiometabolic risk.

### Mechanism 2: Insulin Resistance and Adipocyte Dysfunction

Orexin does not just act in the brain—it has direct effects on the pancreas, adipose tissue, and liver. When that signaling is lost in NT1, the metabolic consequences may go well beyond what weight gain alone can explain.

### Orexin Receptor Expression and Signaling in Metabolic Tissues

Orexin has direct metabolic effects on peripheral tissues that extend beyond its central role. Orexin receptors are expressed in pancreatic islets, adipose tissue, and hepatocytes, allowing orexin signaling to influence metabolism at multiple levels (4).

In pancreatic beta cells, orexin-A enhances beta-cell function and mass through OX1R-mediated activation of phosphoinositide 3-kinase/protein kinase B (PI3K/Akt) and extracellular signal-regulated kinases 1 and 2 (ERK1/2) signaling pathways (6, 15). Notably, these effects are glucose-dependent, meaning orexin potentiates the beta-cell response to ambient glucose rather than independently driving insulin release (15). This pathway is clinically relevant beyond narcolepsy. In oncology, drugs that inhibit the PI3K/Akt/mechanistic target of rapamycin (mTOR) pathway—now a major class of targeted cancer therapies—commonly cause insulin resistance and hyperglycemia. By analogy, loss of orexin-mediated activation of this pathway in NT1 may contribute to insulin resistance and downstream cardiometabolic dysfunction (6). Experimental work using INS-1E cells suggests that orexin-A modulates insulin secretion through calcium-dependent signaling and transient receptor potential channels (15). These effects seem to depend on both receptor subtype and the surrounding metabolic environment.

The insulinotropic effects of orexin-A are glucose-dependent. Orexin-A enhances glucose-stimulated insulin secretion through intracellular calcium mobilization involving adenylate cyclase and ryanodine receptor pathways, with exchange protein directly activated by cyclic adenosine monophosphate 2 (Epac2) playing a role (43). That detail is probably important, since it implies that orexin is more involved in amplifying responses when glucose is already elevated rather than driving insulin secretion at baseline.

Time-course studies suggest that orexin participates in postprandial metabolic regulation, with changes in insulin and leptin levels following glucose loading (43). The effect appears relatively transient, which again fits with a role in short-term metabolic adjustment.

Orexin-A has also been shown to activate the mTOR pathway in beta cells via OX1R, which may support both beta-cell function (including glucose-responsive insulin release) and cell maintenance through promotion of cell growth and survival (16). How much this contributes to long-term beta-cell function is still not entirely clear, but it is a plausible link.

### Adipose and Hepatic Effects

In adipose tissue, orexin-A promotes glucose transporter type 4 (GLUT4) translocation and adiponectin secretion through orexin-1 receptor (OX1R)-mediated activation of peroxisome proliferator-activated receptor gamma/CCAAT/enhancer-binding protein alpha (PPARγ/CCAAT/(C/EBPα) pathways while exerting anti-inflammatory effects via Sirtuin-1/NOD-, LRR-, and pyrin domain-containing protein 3 [sirtuin-1 (SIRT1)/NOD-, LRR-, and pyrin domain-containing protein 3 (NLRP3)] signaling (6). Orexin also enhances energy expenditure through browning of white adipose tissue (44). When orexin signaling is lost, insulin resistance develops at the level of the adipocyte—insulin can no longer suppress hormone-sensitive lipase, leading to increased lipolysis and excess free fatty acid release into the circulation (45). This is the primary driver of the atherogenic dyslipidemia seen in metabolic syndrome [elevated triglycerides, low high-density lipoprotein (HDL), increased small dense low-density lipoprotein (LDL)], and the excess free fatty acids also accumulate ectopically in the liver and muscle, worsening systemic insulin resistance (46).

In the liver, orexin suppresses gluconeogenesis via peroxisome proliferator-activated receptor gamma coactivator 1-alpha (PGC-1α) downregulation and maintains endoplasmic reticulum (ER) homeostasis through daily bidirectional regulation of the autonomic nervous system (6, 47). Orexin-A further activates the hepatic OX1R/PI3K/Akt/mechanistic target of rapamycin complex 1 (mTORC1) pathway, regulating forkhead box protein O1 (FoxO1) phosphorylation and promoting hepatocyte survival (48). Without tonic orexin input, hepatic insulin resistance develops, in which insulin fails to suppress gluconeogenesis while simultaneously driving de novo lipogenesis via sterol regulatory element-binding protein 1c (SREBP-1c), promoting hepatic steatosis. The adipocyte-derived free fatty acid overflow compounds this by overwhelming hepatic lipid disposal. Consistent with this, orexin-deficient mice on a high-fat diet develop severe metabolic dysfunction–associated steatohepatitis (MASH) with fibrosis and hepatocellular carcinoma, driven by unchecked ER stress; daily orexin-A supplementation attenuated these changes (17).

Orexin also contributes to energy expenditure through activation of brown adipose tissue thermogenesis (4, 18). This involves hypothalamic regulation of sympathetic output. Part of this regulation occurs through the melanocortin system—orexin neurons in the lateral hypothalamus project to melanocortin-4 receptor (MC4R)-expressing neurons in the paraventricular nucleus, which in turn drive sympathetic outflow to brown adipose tissue (49). In orexin-null and orexin/ataxin-3 transgenic mice, orexin deficiency is associated with lower energy expenditure, largely due to impaired brown adipose tissue thermogenesis and reduced spontaneous physical activity. As a result, these animals are more prone to weight gain even when caloric intake is similar to, or lower than, that of wild-type controls (18, 19). Loss of orexin thus removes a critical input to the melanocortin-sympathetic-thermogenic axis, reducing energy expenditure independent of food intake (19, 49).

### Evidence of Insulin Resistance

Clinical data suggest that insulin resistance is a central feature of metabolic dysfunction in narcolepsy. Pediatric studies report elevated homeostatic model assessment for insulin resistance (HOMA-IR) in a large proportion of patients, independent of BMI (14). This is notable because it suggests that metabolic abnormalities may develop early and are not simply secondary to changes in body weight.

Meta-analyses further support this, showing elevated insulin levels and increased waist circumference in patients with narcolepsy (7). The magnitude varies across studies, but the overall pattern is fairly consistent.

### Mechanism 3: Autonomic Dysregulation and Disrupted Sleep Architecture

Orexin neurons project to central autonomic structures including the rostral ventrolateral medulla (RVLM), NTS, and raphe pallidus, where they function as a gain controller, modulating sympathetic and parasympathetic output based on behavioral state rather than driving basal tone directly (50).

The clinical picture is nuanced. Microneurography in patients with untreated NT1 shows lower resting muscle sympathetic nerve activity, heart rate, and blood pressure during wakefulness, with values tracking cerebrospinal fluid (CSF) hypocretin-1 levels (24). Yet heart rate variability studies find the opposite during sleep—medication-naïve NT1 patients have consistently higher heart rates across all sleep stages independent of fragmentation, and an elevated LF/HF ratio during non-rapid eye movement (NREM) sleep suggests sympathovagal imbalance (25, 26). What seems to be happening is not a simple shift in one direction but rather a loss of autonomic flexibility—reduced tonic sympathetic drive at rest with inadequate parasympathetic compensation when it matters most (51).

Cardiovascular reflex testing in treatment-naïve patients supports this. Studies have found blunted Valsalva ratios, reduced respiratory sinus arrhythmia, and impaired cold face responses—all pointing to parasympathetic dysfunction that cannot be attributed to medications (24). During sleep, the dissociation is even more striking: sleep-onset rapid eye movement periods (SOREMPs) in NT1 lack the sympathetic surge that normally accompanies rapid eye movement (REM) sleep, instead resembling light NREM (24). In orexin-deficient mice, the nocturnal blood pressure fall is blunted, and REM-associated pressure swings are exaggerated—effects abolished by adrenergic blockade—offering a mechanistic basis for the nondipping pattern seen clinically (50).

In short, orexin deficiency does not simply raise or lower sympathetic tone. It disrupts the ability to appropriately adjust cardiovascular output across changing states—and over time, that loss of flexibility likely adds to cardiovascular risk in NT1 (51, 52).

Narcolepsy is associated with sleep instability. Beyond excessive daytime sleepiness (EDS), patients often have reduced sleep efficiency, more frequent awakenings, and less consolidated slow-wave sleep (20, 21). These changes are generally thought to reflect the loss of orexin-mediated stabilization of sleep-wake states. Without that stabilizing influence, the system transitions easily between states rather than maintaining sustained periods of sleep or wakefulness.

Sleep fragmentation and irregular sleep timing have both been linked to metabolic dysfunction in several large cohort studies. Variability in sleep patterns, even when total sleep time is similar, has been associated with a higher risk of metabolic syndrome and related abnormalities (53, 54). The relationship is not entirely straightforward, but it does seem that disrupted sleep continuity itself—not just sleep duration—plays a role.

Beyond sleep fragmentation, circadian misalignment may further compound metabolic risk. Resting energy expenditure varies with circadian phase, and in shift workers—a well-studied model of circadian disruption—simulated night-shift schedules reduce total daily energy expenditure while increasing acylated ghrelin levels and appetite for energy-dense foods (55–57). Broader evidence suggests that sleep disruption promotes weight gain primarily through increased energy intake driven by alterations in appetite-regulating hormones rather than through large changes in energy expenditure (58).

In narcolepsy specifically, Donjacour et al. measured energy expenditure in patients with NT1 and matched controls using both respiration chamber calorimetry and doubly labeled water and found no significant differences in resting, 24-h, or activity-induced energy expenditure (22). These calorimetric findings suggest that reduced energy expenditure is unlikely to be the primary driver of weight gain in this population, and instead point toward alterations in satiety signaling—consistent with orexin’s known role in appetite regulation—as the more likely contributor to positive energy balance (22, 58).

A related and clinically relevant finding is the loss of nocturnal blood pressure dipping, which is seen in a substantial proportion of patients with NT1 (8). In the general population, nondipping patterns are associated with higher cardiovascular risk, including increased rates of stroke and cardiac events. In narcolepsy, this likely reflects a combination of altered autonomic regulation and the effects of fragmented sleep. Orexin neurons project to key autonomic centers, and their loss may interfere with the normal nocturnal reduction in sympathetic tone (23).

At the same time, repeated arousals and sleep stage transitions may further contribute to sympathetic activation. It is probably not a single mechanism but rather an overlap of several processes that together shift the normal circadian pattern of blood pressure regulation.

### Mechanism 4: Autoimmune Inflammation

Narcolepsy type 1 is widely considered to be an autoimmune condition characterized by selective destruction of hypocretin-producing neurons (3). The strength of the association with human leukocyte antigen DQB1*06:02 (HLA-DQB1*06:02) is striking and has been one of the more consistent findings across studies (3, 27). Additional genetic associations involving T cell receptor loci and other immune-related genes further support this model, although the exact sequence of events is still not completely defined.

Environmental triggers appear to play a role as well. Epidemiological data linking narcolepsy onset to influenza infection and vaccination—particularly during the 2009 H1N1 pandemic—have been difficult to ignore (3, 59). These observations led to the hypothesis that molecular mimicry may be involved, where immune responses directed at viral antigens cross react with hypocretin-related peptides.

Experimental work has provided some support for this idea. Cross-reactive T cell populations recognizing both influenza antigens and hypocretin peptides have been identified, suggesting at least a plausible mechanistic link (27). Animal models have also shown that immune activation in genetically susceptible settings can lead to targeting of orexinergic neurons, involving both CD4^+^ and CD8^+^ T cell responses (59).

Direct evidence in humans comes from studies demonstrating autoreactive T cells targeting hypocretin-associated antigens (28). These cells have been detected in both peripheral blood and cerebrospinal fluid, indicating that they are capable of accessing the central nervous system.

What remains less clear is how much ongoing inflammation persists after the initial neuronal loss. Some postmortem studies show evidence of gliosis in the hypothalamus, suggesting prior inflammatory activity, but whether there is sustained low-grade inflammation contributing to systemic metabolic effects is still somewhat uncertain.

That said, chronic inflammation is already known to be linked to insulin resistance and endothelial dysfunction in other settings. So even if it is not the primary driver, it could still contribute to the overall cardiometabolic profile seen in narcolepsy.

### Metabolomic Alterations and Cardiovascular Risk

The metabolic profile observed in narcolepsy seems to reflect the combined effects of orexin deficiency, sleep disruption, and inflammation rather than any single mechanism. Meta-analytic data show abnormalities in glucose metabolism, lipid parameters, and inflammatory markers (7). These findings are broadly consistent with features of metabolic syndrome, although the pattern is not always uniform across studies.

Large retrospective analyses add to this picture by showing increased risk of hypertension, hyperlipidemia, diabetes, nonalcoholic fatty liver disease, and major adverse cardiovascular events (9). These associations persist even after adjusting for commonly used narcolepsy medications, which suggests that the underlying disease process itself plays a major role.

One of the more notable findings is that these risks are present even in younger individuals, including those under 25 yr of age (9). That observation makes it less likely that these are purely long-term complications and instead points toward early metabolic changes that may accumulate over time.

From a clinical standpoint, this raises the question of whether screening for metabolic abnormalities should be more routine in patients with narcolepsy, even early in the disease course.

## FUTURE DIRECTIONS

### Limitations of Present Therapies

Current treatments for narcolepsy—specifically sodium oxybate, modafinil/armodafanil, solriamfetol, and pitolisant—are effective for symptom control but do not address the underlying orexin deficiency (5). These interventions improve wakefulness or reduce cataplexy, but do not restore the signaling pathways that are lost.

Overall, current therapies are not designed to modify the metabolic aspects of the disease, and there is limited evidence that they do so in practice.

### Orexin Receptor Agonists

The development of selective OX2R agonists represents a different therapeutic approach—one that attempts to replace lost orexin signaling rather than bypass it. The rationale comes from the observation that OX2R plays a dominant role in wake regulation, whereas OX1R appears less critical in this context (5).

TAK-994 was one of the first agents in this class to show strong clinical efficacy. In trials, it led to substantial improvements in wakefulness and reductions in cataplexy frequency (60). However, its development was halted due to hepatotoxicity, and this drug never came to market.

Oveporexton (TAK-861), which is a highly selective oral orexin receptor 2 agonist, is a structurally distinct compound that has shown similar efficacy without the same liver toxicity concerns, at least in early studies (5). Improvements in both objective and subjective measures of sleepiness have been reported, along with reductions in cataplexy. The adverse effect profile—mainly insomnia and urinary symptoms—appears manageable so far, although longer-term data are still pending.

### Potential Metabolic Effects

An open question is whether restoring orexin signaling will translate into measurable improvements in cardiometabolic outcomes in patients with NT1. Given what is known about orexin’s role in insulin sensitivity, adipocyte function, and energy expenditure, it is biologically plausible. Most trials to date have focused on sleep-related endpoints, and metabolic outcomes have not been systematically evaluated; more investigation is needed to better answer this important question.

## CONCLUSIONS

Orexin deficiency in NT1 contributes to cardiometabolic risk through multiple, interconnected pathways, including dysregulated satiety signaling, insulin resistance, sleep disruption, and autoimmune inflammation. These mechanisms are interdependent due to their overlap, thus reinforcing their sequelae and effects.
